# A Review of Biologically Active Natural Products from Mediterranean Wild Edible Plants: Benefits in the Treatment of Obesity and Its Related Disorders

**DOI:** 10.3390/molecules25030649

**Published:** 2020-02-03

**Authors:** Mariangela Marrelli, Giancarlo Statti, Filomena Conforti

**Affiliations:** Department of Pharmacy, Health and Nutritional Sciences, University of Calabria, 87036 Rende (CS), Italy; mariangela.marrelli@unical.it (M.M.); giancarlo.statti@unical.it (G.S.)

**Keywords:** alimurgic plant, biodiversity, obesity, phytochemical compound, wild

## Abstract

Wild foods constitute an essential component of people’s diets around the world. According to the Food and Agriculture Organization (FAO), over 100 million people in the EU consume wild foods, while 65 million collect some form of wild food themselves. The Mediterranean basin is a biodiversity hotspot of wild edible species. Nowadays, due to the renewed interest in alimurgic plants and the recent findings on the beneficial role of their phytochemical constituents, these species have been defined as “new functional foods”. Research on natural products has recently regained importance with the growing understanding of their biological significance. Botanical food supplements marketed for weight and fat loss in obese subjects will be one of the most important items in marketed nutraceuticals. The aim of this report was to review the phytochemical compounds of Mediterranean wild edible species and their therapeutic potential against obesity and its related disorders. Results on the in vitro and in vivo activity of the most interesting plant extracts and their bioactive components are presented and discussed. The most interesting discoveries on their mechanisms of action are reported as well. Overall, this contribution highlights the importance and beneficial health roles of wild edible species.

## 1. Introduction

Plants are a constant and fundamental element of the environment that surrounds us. Until the not too distant past, wild edible plants represented a fundamental food resource for local populations (peasants, woodsmen, shepherds, etc.) during scarcity periods. Since ancient times, spontaneous plants have been commonly used in traditional Mediterranean culture for various purposes, such as food and medicines, but also for the production of clothes and magic and religious rituals. Garn and Leonard suggested that there are between 300,000 and 500,000 plant species on the planet, 30,000 of which are thought to be edible [1], and only 7000 of these have been either cultivated or collected as food. Currently, only 20 species provide 90% of the world’s food requirements, with wheat, maize, and rice being the principal ones. The large-scale cultivation of a limited number of crops, together with the industrial revolution, lifestyle changes, and poor contact with nature, has produced an underutilization of wild dietary plants [2].

Many people, particularly in Mediterranean region, continue to use wild dietary plants in their local popular traditions. The use of spontaneous plants for culinary purpose has always been well rooted in Mediterranean region, although in many cases wild edible plants are known and used only by older people [3]. An unlearning phenomenon has been observed, referring to the situation in which the various uses of plants (in particular those relating to emergency foods) are still remembered but no longer practiced, and are therefore destined to be forgotten [4].

Different trends in the consumption of wild food plants are linked to economic and environmental factors. Some sociocultural factors, such as changes in livelihoods and lifestyles and legal restrictions on the collection of collect wild plants, can help to explain the decrease of wild food plant consumption, not to mention the fact that wild plants are sometime considered famine foods. On the other hand, other sociocultural factors, such as the local appreciation of taste and flavor or the search for natural and healthy foods, seem to be increasing the interest towards wild food plants [5,6]. 

Moreover, in modern societies, a new phenomenon associated with the use of wild dietary plants is emerging. Today, food, and especially food plants, is considered not only in terms of simple nutritional intake, but as a potential source of natural products useful for human health. In many countries, the opportunity has now emerged to elaborate and propose precise indications of dietary behavior to satisfy the needs of the population to preserve their health [7]. One of the opportunities to find solutions to diversify the diet and to increase the intake of nutraceuticals can be found in the popular traditional use of wild edible plants both as a source of food and as compounds with potential therapeutic activity. There is a different sensitivity for healthy eating in different countries that recalls the specificity of the local flora and expresses itself in the local ethnobotanical traditions. Therefore, the study of local popular traditions with nutraceutical activity and their possible reintroduction into modern food diets is of great importance [8]. In many areas of Europe (mainly in Mediterranean countries), there has been a recent revival of interest in the use of dietary wild plants, which are sold in local markets [9]. 

Wild plants usually contain a large spectrum of plant secondary metabolites like polyphenols and terpenoids, which make them good candidates as nutraceuticals, i.e., functional foods. Numerous wild edible plants have been studied in in vitro and in vivo models and have been shown to possess potentially beneficial active principles [10]. Wild-growing plants are used by increasing numbers of people, even by professional chefs, especially in high-end restaurants. They generally are highly accepted by consumers, who often consider that “natural” means “safe”. This is, however, an oversimplification, as numerous botanicals have been found to contain toxic compounds. Therefore, it is essential to provide the general public as well as healthcare professionals with adequate information regarding the benefits and the risks associated with the use of these products, thereby promoting more rational decisions in this regard [11].

## 2. Phytoalimurgy

The branch of botany focused of the rediscovery and the study of wild plants for their nutritional value is called “phytoalimurgy”. This term derives from Greek and Latin words “φυτόν (phytόn)”, which means plant, and “alimenta urgentia”, which indicates the food that can be used in case of necessity and urgency [12]. This word was first coined by the Italian naturalist Giovanni Targioni Tozzetti in 1767, in his work “De alimenti urgentia”, and then by the botanist Oreste Mattirolo, who added the prefix “phyto” to the word in his book “Phytoalimurgia Pedemontana” [13]. Oreste Mattirolo described how European populations have tried to solve the terrible food shortages in wartime, e.g., when wheat was not available, bread was made by mixing different kinds of flour, such as buckwheat and oak acorn flour, which are less nutritious than traditional flour.

The enhancement of the flora and its uses represents a real strategy by which to safeguard biodiversity. Biodiversity, in fact, is not just the number of species present in an area, but the manner in which the plants are grown, and is accompanied by a parallel path of man and plants. It is not by chance that the interest in ethnobotany is growing: saving this knowledge also saves the cultural identity of a population. The folk uses of plants, based on an ancient empirical knowledge, are an expression of the resilient relationship between human communities and their environment, which is influenced by different cultural and socioeconomic changes. The purpose of ethnobotanical investigation is often to select species with which to stimulate pharmacological studies. However, in the context of sustainable land management, the traditional knowledge can also be useful in the improvement of local products by promoting responsible tourism, particularly in nature reserves [14,15]. The alimurgic flora represents a strategic resource to which it is possible to associate many positive agri-food, ecological, and sociocultural values: food source, organic crops, low environmental impact, enhancement of local resources, conservation of biodiversity, conservation of traditional knowledge, income support to medium-sized companies, and introduction into the diet of new species with medicinal and nutraceutical potential [14].

## 3. Obesity and Related Disorders

Obesity is currently one of the main public health concerns since it is a major contributor to the global burden of chronic diseases, including cardiovascular disease, non-alcoholic fatty liver disease, type 2 diabetes mellitus, and certain types of cancer. Obesity is reaching epidemic proportions worldwide, with tripled prevalence in many European countries and affecting a large percentage of the population. Obesity and related disorders are commonly perceived as problems of rich countries, but they are increasingly present even in poor countries [16]. 

Guh and co-workers studied the incidence of comorbidities related to obesity and overweight. Eighteen comorbidities were identified, particularly diabetes, cardiovascular diseases, coronary heart disease, hypertension, colorectal cancer, gallbladder cancer, pancreatic cancer, ovarian cancer, and asthma, from which the authors concluded that overweight and obesity will have a significant impact on healthcare costs [17].

The increasing prevalence of obesity is a major cause of the development of metabolic syndrome, otherwise known as syndrome X. It is a common metabolic disorder that reflects overnutrition and sedentary lifestyles, and is linked to abdominal obesity, insulin resistance and diabetes, blood lipid disorders, inflammation, and increased risk of developing cardiovascular diseases [18,19,20]. Metabolic syndrome has been defined by the WHO as a pathological condition characterized by abdominal obesity, insulin resistance, hypertension, and hyperlipidemia [21].

**Figure 1:**
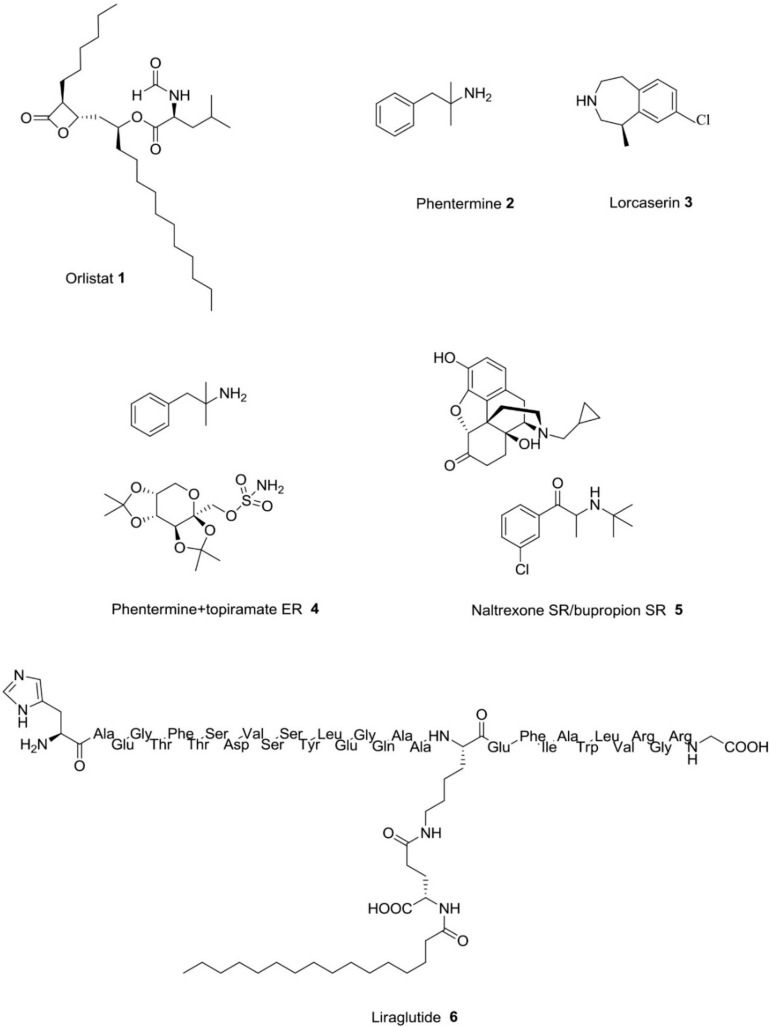
Current commonly used anti-obesity drugs [22,25].

Different potential factors contribute to obesity: genetic background, diet, and physical activity being the major ones. Therefore, the treatment of obesity consists of a reduction of caloric dietary intake combined with an increase in physical activity, while a pharmacological treatment is preferred when the behavioral approach is not sufficient to obtain weight control. In the last few years, different drugs have been promoted but some of them have been successively withdrawn because of their serious adverse effects (dinitrophenol, sibutramine, rimonabant). For many years, orlistat (**1**; Figure 1) was the only commonly used anti-obesity drug approved by the EMEA in Europe for long-term use, while in the USA, besides orlistat, phentermine (**2**) was also available, even if only for short-term use [22]. Orlistat is a semisynthetic hydrogenated derivative of the natural lipase inhibitor produced by *Streptomyces toxytricini* and it is a potent gastrointestinal lipase inhibitor able to prevent dietary fat absorption by 30%, inhibiting both pancreatic and gastric lipases. However, it has various side effects, such as diarrhea, fecal incontinence, flatulence, bloating, and dyspepsia [23,24]. Recently, four other new drugs have been introduced: lorcaserin (**3**) and phentermine+topiramate ER (**4**), approved by the FDA in 2012, and naltrexone SR/bupropion SR (**5**) and liraglutide (**6**), recently approved in both the USA and Europe. However, these new drugs may also cause a number of adverse effects [25].

## 4. In Vitro and In Vivo Effects on Carbohydrate Metabolism

The breakdown of carbohydrate by carbohydrate-digesting enzymes such as α-amylase and α-glucosidase and the subsequent intestinal glucose absorption remain therapeutic targets for the obesity-related disorder diabetes.

**Figure 2:**
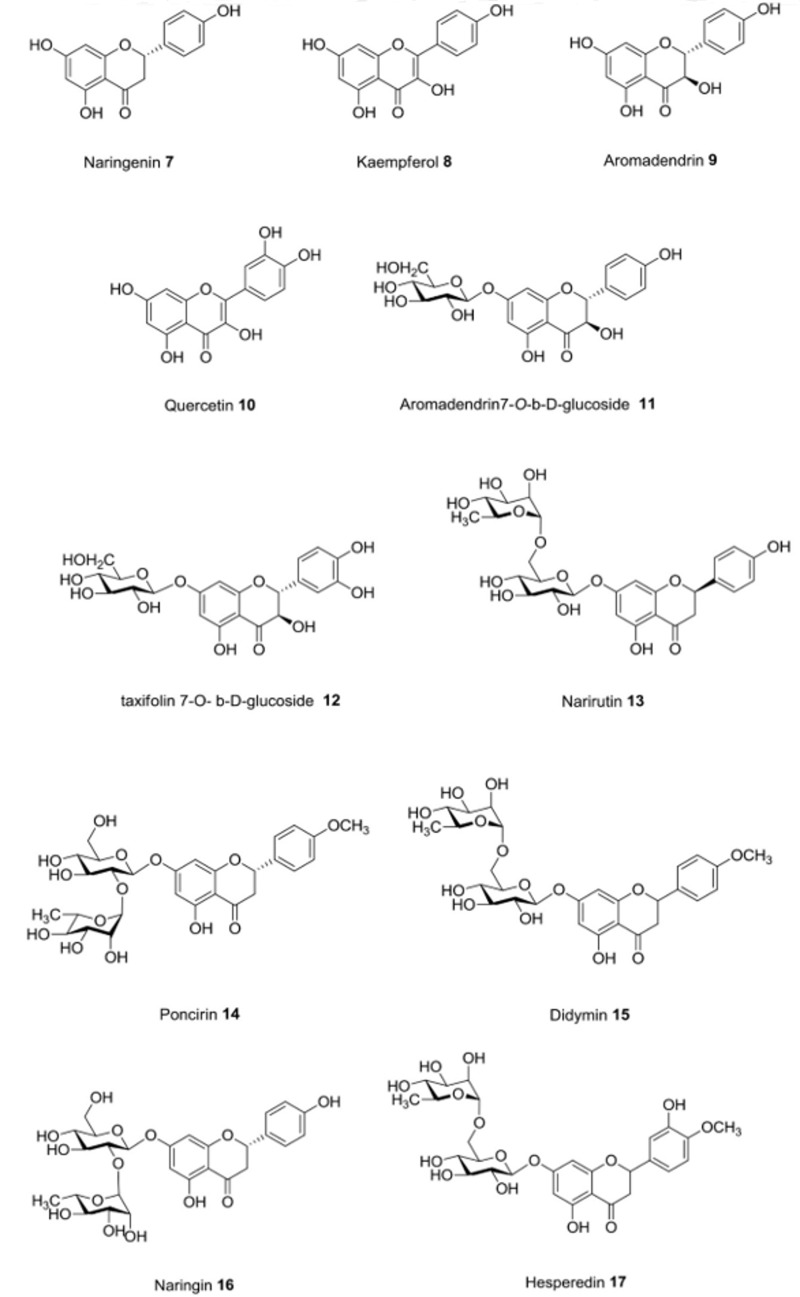

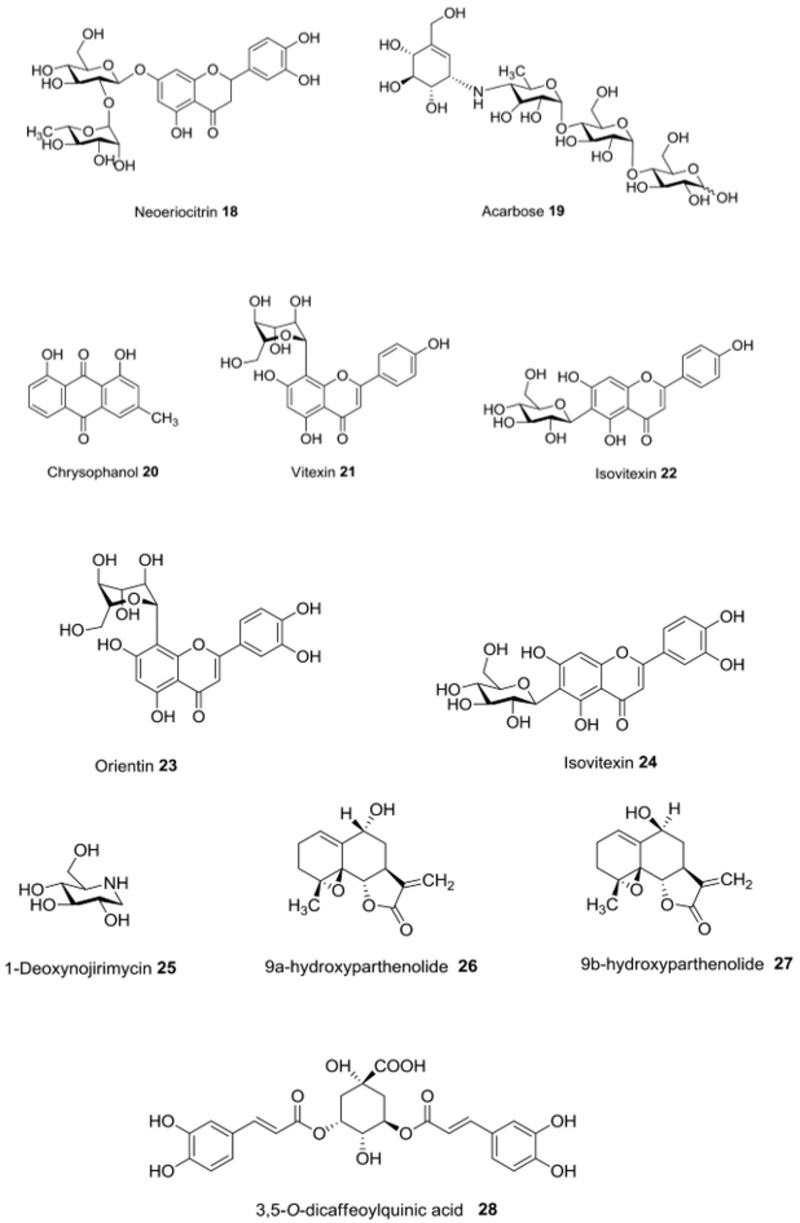
Natural compounds with hypoglycemic properties [26,31,36,40,42,46].

Mehanna and co-workers [26] evaluated the potential anti-obesity effect of the crude extract of *Cuscuta pedicellata* Ledeb. and 10 of its isolated metabolites in high-fat diet (HFD)-fed rats (Table 1). The mechanism of action included the reduction of insulin resistance and glucose tolerance, improving the cellular energy homeostasis and possession of antioxidant activity, with the most active compounds being naringenin (**7**; Figure 2), kaempferol (**8**), aromadendrin (**9**), quercetin (**10**), aromadendrin-7-O-β-D-glucoside **(11**), and taxifolin 7-O-β-d-glucoside (**12**).

Bilberry is a European wild blueberry that contains a higher content of anthocyanins than cultivated blueberry species. Studies in animal models and clinical studies have demonstrated that the supplementation or the consumption of blueberry or blueberry bioactive compounds causes changes in glucose metabolism and improves insulin sensitivity [27]. In particular, the supplementation of 2% freeze-dried blueberry powder for 13 weeks in obese Zucker rats induced significant reductions in glucose, fasting insulin, and insulin resistance [28]. Likewise, Vuong and co-workers [29] showed that blueberry juice fermented by the *Serratia vaccinia* bacterium significantly reduced blood glucose levels and maintained the glycemia of pre-diabetic mice at a normal level. These results indicated that blueberry intake could reduce phenotypes of diabetes in obesity-prone rats by regulating glucose metabolism.

The effects of wild blueberry on blood glucose levels and other parameters involved in glucose metabolism were investigated in obese Zucker rats [30]. Wild blueberries are one of the richest fruit sources of anthocyanins, a class of phenolic bioactive compounds that have been shown to have insulin-sensitizing effects and to improve glucose utilization. In this study, the authors measured fasting plasma concentrations of glucose, insulin, glycated hemoglobin, resistin, and retinol-binding protein 4. The expression of resistin and glucose transporter GLUT4 genes was also determined in the liver and the abdominal adipose tissue. The results showed that plasma HbA1c, retinol-binding protein 4, and resistin concentrations were significantly lower after the administration of wild blueberry. According to these results, regular wild blueberry consumption could potentially help to normalize the dysregulated glucose metabolism associated with, for example, metabolic syndrome.

The hypoglycemic properties of *Poncirus trifoliate* (L.) Raf. juice, seed extracts, and peel essential oil were investigated via inhibition of carbohydrate-hydrolyzing enzymes [31]. All samples were able to inhibit α-amylase and α-glucosidase enzymes in a concentration-dependent manner. The most interesting activity was found against α-glucosidase enzyme. In particular, juice exhibited an IC_50_ value of 81.27 µg/mL, followed by seed extracts (IC_50_ value of 170.54 µg/mL). The same trend was observed against α-amylase, with IC_50_ values of 138.14 and 459.58 µg/mL, respectively, for juice and seed extract. The main flavonoids identified in *P. trifoliata* juice were also investigated for their inhibitory activity. All tested compounds, narirutin (**13**; Figure 2), poncirin (**14**), didymin (**15**), naringin (**16**), hesperidin (**17**), and neoeriocitrin (**18**) demonstrated α-amylase- and α-glucosidase-inhibitory properties in a concentration-dependent manner, being even more active against α-amylase than the positive control acarbose (**19**) (IC_50_ of 4.69–70.80 µM vs. IC_50_ of 77.45 µM). Neoeriocitrin was the most active molecule against α-amylase, with an IC_50_ value of 4.69 µM, while didymin demonstrated the most promising activity against α-glucosidase (IC_50_ = 4.20 µM). 

Several citrus juices are also able to exert hypoglycemic effects. The administration of *Citrus paradisi* Macfad. juice was found to significantly reduce rapid blood glucose levels without any effect on 1.5 h plasma insulin levels in non-diabetic rats [32]. *Citrus medica* L. cv. Diamante (Diamante citron) peel extract administered in ZDF rats exerts a dose-dependent effect on serum glucose levels. The leaves, mesocarp, and endocarp extracts of *C. medica* cv Diamante exerted a moderate carbohydrate-hydrolyzing enzyme inhibition [33,34]. The effects of bergamot (*Citrus bergamia* Risso et Poit.) juice extract on diet-induced hyperlipaemia in Wistar rats and in 237 patients with hyperlipaemia associated or not with hyperglycaemia were demonstrated by Mollace and co-workers [35]. 

Mollica and co-workers evaluated the in vitro enzyme inhibitory activities of extracts of different plant part (flower, stem, and bulb) from *Allium scorodoprosum* L. subsp. *rotundum*, also known as wild garlic [36]. Among tested samples, the stem extract of *A. scorodoprasum* was the most active inhibitor of α-amylase. This extract contained high levels of flavonoids.

Hawash and colleagues evaluated the hydrophilic and lipophilic fractions of seven traditional edible and medicinal wild plants from Palestine for their α-amylase inhibitory activity [37]. The plants in this study included *Arum palaestinum* Boiss., *Malva sylvestris* L., *Plantago major* L., *Centaurea iberica* Trevir. ex Spreng., *Cichorium endivia* L., *Bituminaria bituminosa* (L.) C.H. Stirt, and *Sisymbrium irio* L. All the samples were tested at a concentration of 100 μg/mL. Among the studied hydrophilic fractions, *C. iberica* and *C. endivia* had the highest porcine pancreatic α-amylase-inhibitory effect while those from *S. irio* and *A. palaestinum* were the most active lipophilic fractions.

Melucci and co-workers evaluated the α-amylase and α-glucosidase inhibitory potential of extracts from *Asphodeline lutea* Reichenb., collected in diverse sites in the Italian Central Apennines, at different phenological stages [38]. The results showed that the samples possessed high α-glucosidase-inhibitory effects, particularly the roots of flowering samples. The activity on α-glucosidase is probably due to chrysophanol (**20**), which was identified in all the investigated samples. This anthraquinone has been shown to inhibit mammalian intestinal α-glucosidase activity [39].

In the study of Mzoughi and co-workers [40] the α-amylase- and α-glucosidase-inhibitory activities of ethanol extract from Swiss chard leaves were assessed. The wild Swiss chard (*Beta vulgaris* L. var. cicla) is a glycophytic belonging to the Chenopodiaceae family, distributed all over the world and employed as a green leafy vegetable for its year-round availability and low cost. The leaves ethanol extract induced significant inhibitory effects on α-glucosidase (IC_50_ = 0.13 mg/mL) and α-amylase (IC_50_ = 1.03 mg/mL) activities. The authors attributed the observed enzymes inhibitory effects of the extract to saponins, able to inhibit gluconeogenesis and glycogenolysis. However, other molecular pathways are potentially involved and remain to be investigated. In fact, it was suggested that the enzyme inhibitory capacity of Swiss chard extract might be due to its content of flavonoids, via the inhibition of glucose transporters. Some C-glycosyl flavones, such as vitexin (**21**; Figure 2), isovitexin (**22**), orientin (**23**), and isoorientin (**24**), contained in the leaves and seeds of this species, have been found to inhibit α-glucosidase and might be responsible for the enzyme-inhibitory activity.

The chemical composition and biological activities of different solvent extracts from wild *Cotoneaster nummularia* Fisch. et Mey. were evaluated by Zengin and colleagues [41]. The methanolic extract possessed very good inhibitory activity against α-glucosidase and α-amylase. In this case, the effective inhibitory activities of this sample were also related to their high phenolic content.

Spìnola and co-workers evaluated the ability of *Rubus grandifolius* L. methanolic extracts to inhibit glucosidases (α-, β-), α-amylase, and lipase enzymes [42]. *R. grandifolius* samples, wild berries (fully ripe) and leaves, were collected in two different locations. All the extracts strongly inhibited α-glucosidase, showing lower IC_50_ values than commercial drug acarbose (**19**). Leaves were the most active sample, while berries showed an activity similar to the positive control 1-DNJ (1-deoxynojirimycin) (**25**), an effective natural inhibitor isolated from mulberry roots. 

Five species of edible plants from the Calabria region (southern Italy) were investigated for their α-amylase inhibitory potential: *Borago officinalis* L., *Capparis sicula* Veill., *Echium vulgare* L., *Mentha aquatica* L., and *Raphanus raphanistrum* L. subsp. *raphanistrum* [43]. All the samples were able to inhibit the enzyme, *B. officinalis* being the most active one, with an IC_50_ value of 31.61 μg/mL, which was better than that reported for the widely prescribed drug acarbose, which was used as a positive control. Good inhibitory activity was also found for *E. vulgare* and *C. sicula*, with IC_50_ values equal to 69.18 and 72.85 μg/mL, respectively.

Five other Mediterranean dietary plants from Calabria (*Carduus pycnocephalus* L., *Clematis vitalba* L., *Lepidium sativum* L., *Malva sylvestris* L., and *Picris hieracioides* L.) were also evaluated for their α-amylase inhibitory activity [44]. The formulation obtained from *C. vitalba* exhibited the strongest inhibitory effect on α-amylase (IC_50_ = 31.52 μg/mL). *C. vitalba* extract presented a higher content in fats, mainly due to a single component. In the same extract, the content of phenols was also clearly higher [45], which could have been associated with the recorded high activity. 

Saoud and co-workers tested the anti-α-glucosidase activities of the ethyl acetate (EtOAc) and n-butanol (n-BuOH) extracts from *Anvillea radiata* Coss. & Dur. (Asteraceae). Three isolated compounds were tested as well: two epimer germacranolides, 9α-hydroxyparthenolide (**26**; Figure 2) and 9β-hydroxyparthenolide (**27**), and the phenolic acid 3,5-O-dicaffeoylquinic acid (**28**) [46]. The ethyl acetate and n-butanol extracts inhibited the enzyme with IC_50_ values of 0.92 mg/mL and 0.81mg/mL, respectively. Among the isolated compounds, compound **28** displayed the highest α-glucosidase-inhibitory activity (IC_50_ = 0.09 mg/mL).

## 5. Pancreatic Lipase Inhibition

Lipids represent an important ingredient in human nutrition. They are essential compounds for all living organisms. Lipids are the building blocks of cellular membranes, thermal isolators, and constitute a source and reserve of body energy. However, their long-term increased intake contributes to the development of obesity and is associated with important comorbidities [47]. Agents that inhibit fat digestion can exert beneficial effects in the treatment of obesity [48]. Inhibition of the digestion of dietary lipids is a logical target for a pharmacological intervention, since it does not involve a central mechanism of action [49]. Pancreatic lipase (triacylglycerol acylhydrolase) is a key enzyme for the absorption of dietary triglycerides. Therefore, its inhibition affects fat hydrolysis and leads to a decrease of fat absorption. 

Eighteen spontaneous edible plants gathered in the wild and used in the local folk tradition in the Calabria region of southern Italy, were investigated for their pancreatic lipase inhibition: *Amaranthus retroflexus* L., *Anchusa azurea* Mill., *Asparagus acutifolius* L., *Cichorium intybus* L., *Diplotaxis tenuifolia* (L.) DC., *Foeniculum vulgare* Miller subsp. *piperitum* (Ucria) Coutinho, *Mentha spicata* L. ssp. *glabrata* (Lej. et Court.) Lebeau, *Origanum vulgare* L. subsp. *viridulum* (Martin-Donos) Nyman, *Papaver rhoeas* L. subsp. *rhoeas*, *Portulaca oleracea* L., *Raphanus raphanistrum* L. subsp. *landra* (DC.) Bonnier & Layens, *Rosmarinus officinalis* L., *Rubus caesius* L., *Rumex conglomeratus* Murray, *Silene vulgaris* (Moench) Garcke, *Smyrnium olusatrum* L., *Sonchus asper* (L.) Hill., and *Sonchus oleraceus* L. [50]. Nine extracts showed an IC_50_ value of less than 10 mg/mL. The aqueous ethanol extracts of *P. oleracea* and *S. vulgaris* leaves showed the highest inhibitory potential, with IC_50_ values of 5.48 mg/mL and 6.02 mg/mL, respectively. Moreover, it was observed that among plant species belonging to Lamiaceae, *M. spicata* and *R. officinalis* showed inhibitory activity with IC_50_ values of 7.85 mg/mL and 7.00 mg/mL, respectively. The most interesting species belonging to Asteraceae and Brassicaceae were, respectively, *S. oleraceus* (IC_50_ = 9.75 mg/mL) and *D. tenuifolia* (IC_50_ = 7.76 mg/mL). 

**Figure 3:**
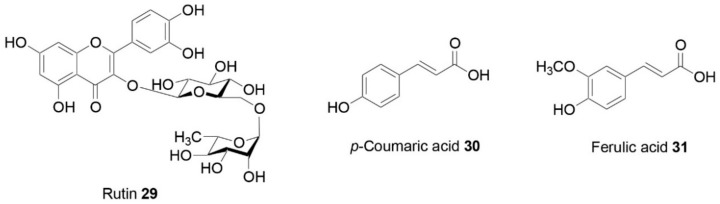
Phytochemicals with pancreatic-lipase-inhibitory properties [54,58].

The inhibition of pancreatic lipase was also evaluated for other five edible plant species from Calabria (Italy): *Borago officinalis* L., *Capparis sicula* Veill., *Echium vulgare* L., *Mentha aquatica* L., and *Raphanus raphanistrum* L. subsp. *raphanistrum* [43]. The best pancreatic lipase inhibitory activity was reported for *C. sicula* hydroalcoholic extract, with an IC_50_ value of 0.5 ± 0.03 mg/mL. *E. vulgare* also showed effectiveness, although to a lesser extent, inducing a percentage of inhibition of 40.54 ± 1.74 (mean ± SD, n = 3) at a concentration of 2.5 mg/mL. *Capparis* sect. Capparis has its maximum diversity in the Mediterranean region [51]. It has been reported that the phytochemical composition of *Capparis* species depends on ecological conditions at the site of collection (soil, exposition, water regime, etc.) [52]. Conforti and co-workers showed the biovariability of different caper species growing wild in Calabria through the detection, isolation, and quantitative evaluation of phytosterols and vitamin E as determined by GC/MS analysis [53]. A more detailed investigation on *Capparis* pancreatic lipase inhibitory activity was realized by Marrelli and co-workers [54]. Twenty accessions of the two species, *Capparis orientalis* Veill. and *C. sicula* Veill. ssp. *sicula* growing wild in Calabria (southern Italy) were characterized with high-performance thin-layer chromatography (HPTLC) analyses through the detection and quantitative evaluation of rutin (**29**, Figure 3) as a typical chemical marker. The lipase-inhibitory potential of the two main metabolites, rutin and glucocapparin, was evaluated as well. All *Capparis* samples affected the enzyme activity in a concentration-dependent manner. IC_50_ values ranging from 0.73 to 2.32 mg/mL were obtained, *C. sicula*, accession no. C6 (rutin content = 25.77 mg/g of plant material), being the most effective sample. Very good activity was also observed for *C. sicula* no. C3, C5, C6, C8, C9, and C15, and *C. orientalis* no. C10, with IC_50_ values ranging from 0.83 to 0.96 mg/mL. The biological activity of the two chemical markers, rutin (**29**) and glucocapparin, was also estimated. Glucocapparin did not induce inhibitory effects, while a very important activity was observed for rutin (IC_50_ = 0.10 mg/mL). 

*Carduus pycnocephalus* L., *Clematis vitalba* L., *Lepidium sativum* L., *Malva sylvestris* L., and *Picris hieracioides* L. were also investigated in order to find enzyme inhibitors [44]. These wild collected food plants are used raw, as salad (*L. sativum*, *P. hieracioides*), boiled (*M. sylvestris*, *C. pycnocephalus*), or fried (*C. vitalba*, *C. pycnocephalus*) in southern Italy. *C. vitalba* extract showed the best inhibitory activity with an IC_50_ value of 0.99 ± 0.18 mg/mL. A very good result was also obtained for *L. sativum* (IC_50_ = 1.28 ± 0.29 mg/mL). *C. vitalba* extract presented a higher content of fatty acids and phenols [45]. This extract mainly constituted chlorogenic acid, (±)-catechin, and caffeic acid. 

Wild *Leopoldia comosa* (L.) Parl. (syn. *Muscari comosum* (L.) Mill.) bulbs were also investigated for their inhibitory activity on pancreatic lipase. The wild bulbs were compared with the same cultivated species that are commonly commercialized, in order to identify the samples with the best quality for potential therapeutic applications [55]. The hydroalcoholic extracts and the ethyl acetate fractions of wild bulbs showed a very important biological activity, with IC_50_ values equal to 0.166 ± 0.005 and 0.153 ± 0.005 mg/mL, respectively. Conversely, the polar fraction obtained from cultivated bulb hydroalcoholic extracts showed a significantly lower activity (IC_50_ = 0.469 ± 0.023 mg/mL), and the raw extract from cultivated samples was not effective at all. Furthermore, wild bulbs showed a phenolic content of 264.33 mg/g while cultivated ones showed a phenolic content more than six times lower (42 mg/g). 

The cooked bulbs of this plant were also investigated. Three treatments were considered: bulbs boiled in water for 15 min (traditional cooking method); bulbs steam-cooked for 15 min (alternative cooking method), and uncooked raw bulbs [56]. Untreated raw bulbs showed the best lipase inhibitory activity compared to steamed and boiled bulbs.

Moreover, *L. comosa* aerial parts (leaves and inflorescences) were also evaluated for both their phytochemical content and their biological activity [57]. The best pancreatic lipase inhibitory potential was demonstrated by the n-hexane and the ethyl acetate fractions of the leaves, with IC_50_ values of 0.369 ± 0.020 and 0.336 ± 0.007 mg/mL. The same fractions from the inflorescence sample were also effective (IC_50_ values equal to 0.736 ± 0.045 and 0.780 ± 0.009 mg/mL, respectively). The two dichloromethane fractions induced a lower but still interesting inhibitory activity. The pancreatic lipase inhibitory activity observed for the EtOAc fractions could to a certain extent have been related to the presence of the flavonoid glycoside rutin (**29**), detected in both leaves and inflorescence ethyl acetate samples. The lipase inhibitory activity exerted by the inflorescence dichloromethane fraction could have been related to the presence of the identified compounds p-coumaric acid (**30**) and ferulic acid (**31**, Figure 3), two phenolic acids known for their ability to inhibit pancreatic lipase [58].

## 6. Hypolipidemic Activity

Diets enriched with blueberries have been reported to improve dyslipidemia. Plasma triglycerides (TG) and total cholesterol (TC) concentrations were significantly reduced in obese Zucker rats supplemented with 8% wild blueberry for 8 weeks [59] or 2% blueberry powder for 13 weeks compared to the control groups [28]. A reduction in serum TC and low density lipoprotein cholesterol (LDL-C) was observed, as well as in the levels of liver TG and TC following the consumption of blueberry juice. However, the contents of liver lipids and cholesterol were not changed in C57BL/6 mice [60]. The consumption of 1%, 2%, and 4% blueberry supplements for 8 weeks significantly reduced the TC and LDL-C concentrations in pigs [61]. The possible pathways involved in the anti-dyslipidemic effect of blueberries include the regulation and expression of key enzymes such as lipoprotein lipase (LPL) [62], fatty acid synthase [63], and ATP-binding cassette transporter 1 (ABCA1) [64], which are involved in TG and cholesterol metabolism.

Del Bo’ and co-workers investigated anthocyanin- and phenolic-acid-rich fractions from wild blueberry for their capacity to counteract lipid accumulation in macrophages derived from monocytic THP-1 cells [65]. Lipid accumulation was reduced at all tested concentrations of the anthocyanins-rich fraction, with 10 μg/mL being the most effective concentration (−27.4%). The phenolic-acid-rich fraction significantly reduced the lipid accumulation only at the lower concentrations (ranging from 0.05 to 0.3 μg/mL). 

## 7. Inhibition of Adipogenesis

When the intake of energy chronically exceeds energy expenditure, most excess energy is stored in the form of triglycerides in adipose tissue. Increased adipose tissue mass can arise through an increase in cell size, cell number, or both. The molecular mechanisms that regulate pre-adipose cell growth, adipose differentiation, and lipogenesis in fat cells have been extensively investigated [66]. Many studies have reported that obesity may induce oxidative stress [66,67]. Pre-adipocyte differentiation has become an area of intense research in recent years and has been primarily studied using in vitro models of adipogenesis, including the 3T3-L1 cell line [68,69]. 

*Oxycoccus quadripetalus* Schinz & Thell., known as wild cranberry (syn. Vaccinium oxycoccos L.) is a good source of bioactive substances with high antioxidant potential and well documented beneficial properties. It contains vitamins, microelements, organic acids, and polyphenols such as flavonoids (quercetin, kaempferol, anthocyanin etc.). A lyophilized cranberry extract was investigated to understand its effects on precursor and mature adipocytes. Its ability to affect lipogenesis, lipolysis, and the expression of adipogenic transcription factors (PPARγ, C/EBPα and SREBP1) has also been verified [70]. Cranberries reduced the proliferation and the viability of 3T3-L1 pre-adipocytes in a dose-dependent manner. Both the number of adipocytes and the lipid accumulation in maturing 3T3-L1 preadipocytes were decreased. Moreover, cranberries directly induced lipolysis in adipocytes and downregulated the expression of transcription factors of the adipogenesis pathway, such as peroxisome proliferator activated receptor gamma (PPARγ), CCAAT/enhancer binding protein alfa (C/EBPα), and sterol regulatory element binding protein 1 (SREBP1).

## 8. Brown Adipose Tissue Activation

**Figure 4:**
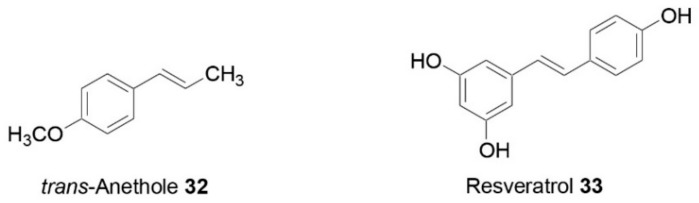
Phytochemical compounds acting by brown adipose tissue activation [73,74].

Both the suppression of white adipose tissue (WAT) expansion and the activation of brown adipose tissue (BAT) are interesting novel potential strategies against obesity. The search for natural compounds for the browning of white adipocytes continues to attract the attention of researchers due to the possibility to find safe and novel tools against obesity [71,72]. Plant-derived essential oils (particularly spices) are a rich source of volatile compounds, and some of them, such as trans-anethole (**32**, Figure 4), have been demonstrated to have anti-obesity properties. In a study by Kang and co-workers, a slight intake of 100 mg/kg of trans-anethole was able to reduce body weight gain in diet-induced obese mice [73]. This molecule acts through the elevation of the expression of beige-specific genes such as Ppargc1α, Cited1, Prdm16, Tbx1, Ucp1, Cd137, and Tmem26. It also regulated lipid metabolism in white adipocytes via adipogenesis and lipogenesis reduction as well as lipolysis and fat oxidation elevation. Furthermore, trans-anethole showed thermogenic activity through the increase of mitochondrial biogenesis in white adipocytes and the activation of brown adipocytes. 

Resveratrol (**33**), a natural phenol and phytoalexin produced by several plants, has been demonstrated to reduce adipose tissue inflammation, causing a decreased expression of the pro-inflammatory cytokines TNF-α and IL-6 in adipose tissue. This compound is also able to increase the expression of genes associated with the “browning” of adipose tissue, including UCP1 and PGC-1α [74].

**Table 1:** Extracts from Mediterranean wild edible plants with beneficial effects against obesity and related comorbidities.

Plant Species	Study	Activity	Class/Bioactive Compounds	References
*Allium scorodoprosum* L. subsp. *rotundum*	In vitro	α-Amylase-inhibitory activity		[36]
*Amaranthus retroflexus* L.	In vitro	Pancreatic lipase inhibition		[50]
*Anchusa azurea* Mill.	In vitro	Pancreatic lipase inhibition		[50]
*Anvillea radiata* Coss. & Dur.	In vitro	α-Glucosidase-inhibitory activity	9α-hydroxyparthenolide (**26**), 9β-hydroxyparthenolide (**27**) and 3,5-*O*-dicaffeoylquinic acid (**28**)	[46]
*Arum palaestinum* Boiss.	In vitro	α-Amylase-inhibitory activity		[37]
*Asparagus acutifolius* L.	In vitro	Pancreatic lipase inhibition		[50]
*Asphodeline lutea* Reichenb.	In vitro	α-Glucosidase-inhibitory effects	Chrysophanol (**20**)	[38,39]
*Beta vulgaris* L.	In vitro	α-Amylase-inhibitory activity; α-glucosidase-inhibitory activity	Vitexin (21), isovitexin (22), orientin (23) and isoorientin (24)	[40]
*Bituminaria bituminosa* (L.) C.H.Stirt	In vitro	α-Amylase-inhibitory activity		[37]
*Borago officinalis* L.	In vitro	α-Amylase-inhibitory activity; pancreatic-lipase-inhibitory activity		[43]
*Capparis orientalis* Veill.	In vitro	Pancreatic-lipase-inhibitory activity	Rutin (**29**)	[54]
*Capparis sicula* Veill.	In vitro	α-Amylase-inhibitory activity; pancreatic-lipase-inhibitory activity	Rutin (**29**)	[43,54]
*Carduus pycnocephalus* L.	In vitro	α-Amylase-inhibitory activity; pancreatic-lipase-inhibitory activity		[44]
*Centaurea iberica* Trevir. ex Spreng	In vitro	α-Amylase-inhibitory activity		[37]
*Cichorium endivia* L.	In vitro	α-Amylase-inhibitory activity		[37]
*Cichorium intybus* L.	In vitro	Pancreatic lipase inhibition		[50]
*Citrus bergamia* Risso et Poit. (bergamot)	In vivo Clinical	Effectiveness against hyperlipaemia associated or not with hyperglycaemia		[35]
*Citrus medica* L. cv. Diamante (Diamante citron)	In vitro	Moderate carbohydrate-hydrolyzing enzyme inhibition;		[33]
	In vivo	Dose-dependent effect on serum glucose levels in ZDF rats		[34]
*Citrus paradisi* Macfad.	In vivo	Rapid blood glucose reduction		[32]
*Clematis vitalba* L.	In vitro	α-Amylase-inhibitory activity; pancreatic-lipase-inhibitory activity		[44]
*Cotoneaster nummularia* Fisch. et Mey	In vitro	α-Amylase inhibitory activity; α-glucosidase-inhibitory activity		[41]
*Cuscuta pedicellata* Ledeb.	In vivo	Reduction of insulin resistance and glucose tolerance	Naringenin (**7**), kaempferol (**8**), aromadenderin (9), quercetin (**10**), aromadenderin-7-*O*-b-d- glucoside (**11**), taxifolin 7-*O*-b-d-glucoside (**12**)	[26]
*Diplotaxis tenuifolia* (L.) DC.	In vitro	Pancreatic lipase inhibition		[50]
*Echium vulgare* L.	In vitro	α-Amylase-inhibitory activity; pancreatic-lipase-inhibitory activity		[43]
*Foeniculum vulgare Miller subsp. piperitum* (Ucria) Coutinho	In vitro	Pancreatic lipase inhibition		[50]
*Leopoldia comosa* (L.) Parl. (syn. *Muscari comosum* (L.) Mill.)	In vitro	Pancreatic lipase inhibition		[55,56,57]
*Lepidium sativum* L.	In vitro	α-Amylase-inhibitory activity; pancreatic-lipase-inhibitory activity		[44]
*Malva sylvestris* L.	In vitro	α-Amylase-inhibitory activity; pancreatic-lipase-inhibitory activity		[37,44]
*Mentha aquatica* L.	In vitro	α-Amylase-inhibitory activity; pancreatic-lipase-inhibitory activity		[43]
*Mentha spicata L*. ssp *glabrata* (Lej. et Court.) Lebeau	In vitro	Pancreatic lipase inhibition		[50]
*Origanum vulgare* L. subsp. *viridulum* (Martin-Donos) Nyman	In vitro	Pancreatic lipase inhibition		[50]
*Oxycoccus quadripetalus* Schinz & Thell. (*syn. Vaccinium oxycoccos* L., wild cranberry)	In vitro	Inhibition of adipogenesis		[70]
*Papaver rhoeas* L. subsp. *rhoeas*	In vitro	Pancreatic lipase inhibition		[50]
*Picris hieracioides* L.	In vitro	α-Amylase-inhibitory activity; pancreatic-lipase-inhibitory activity		[44]
*Plantago major* L.	In vitro	α-Amylase-inhibitory activity		[37]
*Poncirus trifoliata* (L.) Raf.	In vitro	Inhibition of carbohydrate-hydrolyzing enzymes	Nanirutin (**13**), poncirin (**14**), didymin (**15**), naringin (**16**), hesperidin (**17**), neoeriocitrin (**18**)	[31]
*Portulaca oleracea* L.	In vitro	Pancreatic lipase inhibition		[50]
*Raphanus raphanistrum* L. subsp. *landra* (DC.) Bonnier & Layens	In vitro	Pancreatic lipase inhibition		[50]
*Raphanus raphanistrum* L. subsp. *raphanistrum*	In vitro	α-Amylase-inhibitory activity; pancreatic-lipase-inhibitory activity		[43]
*Rosmarinus officinalis* L.	In vitro	Pancreatic lipase inhibition		[50]
*Rubus caesius* L.	In vitro	Pancreatic lipase inhibition		[50]
*Rubus grandifolius* L.	In vitro	Glucosidases (α-, β-), α-amylase, and lipase enzyme inhibition		[42]
*Rumex conglomeratus* Murray	In vitro	Pancreatic lipase inhibition		[50]
*Silene vulgaris* (Moench) Garcke	In vitro	Pancreatic lipase inhibition		[50]
*Sisymbrium irio* L.	In vitro	α-Amylase-inhibitory activity		[37]
*Smyrnium olusatrum* L.	In vitro	Pancreatic lipase inhibition		[50]
*Sonchus asper* (L.) Hill.	In vitro	Pancreatic lipase inhibition		[50]
*Sonchus oleraceus* L.	In vitro	Pancreatic lipase inhibition		[50]
*Vaccinium angustifolium* Ait. (Blueberry)	In vitro	Reduced lipid accumulation in macrophages;		[65]
	In vivo	Lowering of plasma HbA1c, retinol-binding protein 4, and resistin;	Antocyanins	[30]
		Reduction of blood glucose levels;		[29]
		Lowering of plasma TG, TC, and LDL-C concentrations		[28,59,61]
		- Reduction in glucose, fasting insulin and insulin resistance		[28]

## 9. Conclusions

The Mediterranean diet, typical of the regions closest to the Mediterranean Sea, is considered a model of healthy eating for its contribution to a healthy status and a better quality of life. In 2013, the Mediterranean diet was inscribed on the UNESCO Representative List of the Intangible Cultural Heritage of Humanity for Italy, Spain, Portugal, Greece, Cyprus, Croatia, and Morocco [75]. 

This healthy diet model is based on the consumption of abundant and variable plant foods, cereals, and olive oil as the main fat. Such a dietary pattern allows a balanced ratio of n-6/n-3 essential fatty acids, and high amounts of fiber, antioxidants, and vitamins C and E [76]. 

It has been widely demonstrated that adherence to the Mediterranean diet exerts a significant protective effect against the major chronic degenerative diseases [77,78]. Moreover, it has been associated with a lower cancer mortality and risk of several cancer types, especially colorectal cancer [79]. 

The prevalence of obesity is increasing to an alarming level worldwide, and diabetes, one of obesity major comorbidities, is following the same trend. In recent years, a number of epidemiological studies have focused on the relationship between the Mediterranean diet and weight, and most of them demonstrated that the Mediterranean diet is significantly related to a decreased overweight incidence [80,81,82]. 

In the last decade, a huge number of plant extracts and their phytochemical constituents have been investigated to examine their potential anti-obesity effectiveness [83,84]. 

As discussed in the present review, a number of these plants belong to the Mediterranean basin.

In particular, 31 of the reviewed plant species demonstrated inhibitory potential against the pancreatic lipase enzyme. Three bioactive compounds, the flavonoid glycoside rutin and the phenolic acids p-coumaric and ferulic acids, have been proven to be effective in inhibiting this enzyme. It has also been demonstrated that two phytochemical compounds, trans-anethole and resveratrol, act through the activation of the brown adipose tissue. Moreover, wild cranberries have been demonstrated to affect adipogenesis, and a very interesting hypolipidemic activity has been observed for blueberries. 

Finally, interesting effects on carbohydrate metabolism have been reported for 28 plants from Mediterranean basin and for 21 bioactive compounds isolated from these species. 

Overall, numerous studies in the literature have dealt with plant extracts’ and pure compounds’ inhibitory activity against digestive enzymes linked to obesity and diabetes (α-amylase, α-glucosidase, and pancreatic lipase). Other interesting novel potential strategies against obesity have been not fully explored, such as regarding the potential effectiveness of Mediterranean wild edible plants. 

Furthermore, most of the studies reported herein were performed in vitro or in animal models. Thus, further studies are needed to corroborate these initial first results and to understand the underlying mechanisms of action, the long-term safety, and the potential toxicological effects of these plant species and their components. Such studies are needed to find new safe and effective pharmacological treatments for obesity.

## Abbreviations

The following abbreviations have been used in the manuscript:

ABCA1	ATP-binding cassette transporter 1
C/EBPα	CCAAT/enhancer binding protein alfa
EMEA	European Agency for the Evaluation of Medicinal Products
FAO	Food and Agriculture Organization
FDA	Food and Drug Administration
GLUT4	Glucose transporter type 4
HbA1c	Glycated hemoglobin A1c
HFD	High-fat diet
IL-6	Interleukin 6
LDL-C	Low density lipoprotein cholesterol
LPL	Lipoprotein lipase
PPARγ	Peroxisome proliferator activated receptor gamma
SREBP1	Sterol regulatory element binding protein 1
TC	Total cholesterol
TG	Plasma triglycerides
TNF-α	Tumor necrosis factor α
UNESCO	United Nations Educational, Scientific and Cultural Organization
ZDF	Zucker diabetic fatty
